# Evaluation of the Mineral Concentration in Beef from Polish Native Cattle

**DOI:** 10.1007/s12011-015-0549-3

**Published:** 2015-10-26

**Authors:** Piotr Domaradzki, Mariusz Florek, Agnieszka Staszowska, Zygmunt Litwińczuk

**Affiliations:** Department of Commodity Science and Processing of Animal Raw Materials, University of Life Sciences in Lublin, Akademicka 13, 20-950 Lublin, Poland; Department of Breeding and Conservation of Genetic Resources of Cattle, University of Life Sciences in Lublin, Akademicka 13, 20-950 Lublin, Poland

**Keywords:** Beef, Minerals, Polish native cattle breeds, Polish Red, White-Backed, Polish Black-and-White, Simmental, Polish Holstein-Friesian

## Abstract

The aim of the study was to determine the content of macrominerals and microminerals in the *longissimus lumborum* (LL) and *semitendinosus* (ST) muscles of young bulls of five breeds—Polish Red (PR), White-Backed (WB), Polish Black-and-White (PBW), Simmental (SIM) and Polish Holstein-Friesian (PHF). The meat of the Polish Holstein-Friesian bulls was found to contain significantly less K, Mg and Ca and more Mn than other breeds. The meat of the White-Backed bulls contained significantly (*P* < 0.01) more Ca and less Na than the meat of the Polish Red, Simmental and Polish Holstein-Friesian breeds. Moreover, the meat of White-Backed bulls showed a significantly (*P* < 0.01) higher level of Mn and Cu in comparison with other native breeds (Polish Red and Polish Black-and-White) and the Simmental breed. Similar content of macronutrients was found in the LL and ST muscles. However, in the case of micronutrients, the LL muscle contained significantly more Zn, Fe and Cu as well as Mn (*P* > 0.05).

## Introduction

Red meat (mainly beef) is an important source of minerals in the human diet, supplying essential elements with high availability, particularly Fe and Zn [1–4]. The bioavailability of trace elements from beef ranges from 30 to 45 % for Cu, from 40 to 68 % for Zn, from 55 to 95 % for Mn and from 60 to 70 % for Fe [5]. Iron in meat occurs mainly in its easily available haem form, which is bound with myoglobin and haemoglobin. Its absorption from meat (from 20 to 30 %) is nearly twice as high as absorption from plants, although in the case of plants consumed together with meat, iron absorption can even double. Similarly, absorption of zinc from a diet rich in animal protein is greater than in the case of foods of plant origin [6].

In Poland, as in many countries of Central Europe, the most beef is obtained from cattle breeds used for dairy purposes. This, however, is a varied population, ranging from typically dairy cows (Holstein-Friesian, Jersey) to small populations of native breeds, i.e. those occurring in only one country. All four of the Polish native cattle breeds (Polish Red, White-Backed, Polish Black-and White and Polish Red-and-White) are kept on low-input farms where livestock is raised extensively, i.e. pastured in the summer and fed on-farm fodder (silage and hay) in the winter [7]. This type of production system is a key factor determining the high nutritional value of the meat obtained [8]. It is worth emphasising that the literature contains no reports on the mineral concentrations in the meat of Polish native cattle breeds.

The aim of the study was to compare the content of macro- and microminerals in meat of young bulls of five breeds including three natives—Polish Red, White-Backed, Polish Black-and-White, as well as Simmental and Polish Holstein-Friesian. Additionally, the effect of muscle on mineral concentration was estimated.

## Material and Methods

### Samples

The research material consisted of samples of the *longissimus lumborum* (LL, *n* = 40) and *semitendinosus* (ST, *n* = 40) muscles collected from the carcasses of young bulls of five breeds (eight from each breed). The calves included in the experiment were purchased in southeastern Poland from local breeders. During the initial period, calves were fed with milk and milk substitutes and subsequently grass forage, hay and concentrate (up to 6 months of age). The mean body mass of the calves at 6 months of the Polish Red (PR), White-Backed (WB) Polish Black-and-White (PBW) Polish Holstein-Friesian (PHF) and Simmental (SIM) breed were 178.8, 190.6, 199.8, 179.1 and 167.9 kg, respectively.

After this period, the semi-intensive fattening was initiated and continued for 12 months, including both a winter and a summer feeding season. The animals were housed in a tie-stall system. The basic feed during the winter consisted of hay (47 % DM) and maize silage (42 % DM) and in the summer mainly grass forage (49 %) supplemented with maize silage (20 %) and hay (19 %). The feed rations were supplemented with small amounts of grain meal (about 11–12 %). The bulls were slaughtered at an average age of 18 months. The mean body mass of bulls at slaughter and range of carcass conformation and fatness classes (according to the EUROP scale) was 506 kg, R^−^-O^0^, 2^−^-2^+^ for PR; 563 kg, R^−^-O^0^, 2^−^-3^0^ for WB; 593 kg, R^−^-O^0^, 2^−^-3^0^ for PBW; 532 kg, R^−^-O^0^, 2^−^-3^0^ for PHF; and 532 kg, U^0^-O^0^, 2^−^-3^−^ for SIM, respectively.

Muscle samples were collected during dissection of the right half of the carcasses (following 24-h refrigeration at 2 °C, relative humidity 85 %), vacuum-packed in PA/PE vacuum bags and stored at 2–4 °C until analysis, 48 h after slaughter.

### Sample Preparation and Analysis

The muscle samples (1 g) were wet-digested with 9 ml concentrated nitric acid using a MarsXpress microwave oven (CEM Corporation, Matthews, NC, USA). Digested samples were transferred to polypropylene tubes and diluted to 25 ml with ultrapure water. A blank digest (9 ml HNO_3_) was carried out in the same way. The concentration of macrominerals (potassium, sodium, calcium and magnesium) and microminerals (zinc, iron, manganese and copper) was determined by means of flame atomic absorption spectrometry (FAAS; air-acetylene flame) using a Varian Spectra 240FS spectrometer.

In order to determine sodium and potassium levels, a solution of caesium chloride was added as a deionised buffer to all of the samples and standards. A lanthanum chloride solution was used as a correction buffer to determine calcium and potassium. During the analysis, deuterium background correction was used and limits of quantification (LOQ) and detection (LOD) were taken into account. The method accuracy was evaluated using minerals determined in the Standard Reference Material 1577c Bovine Liver. The limits of detection, certified and measured value as well as recovery are presented in Table 1. The analyses were performed in triplicate. The content of macro- and microminerals in the samples was expressed in milligram per kilogram wet mass.

**Table 1:** The detection limits (LOD), minerals concentrations and recoveries (mean ± standard deviation)

	LOD (mg/kg)	Standard reference material 1577c Bovine Liver		Recovery (%)
		Certified value (mg/kg)	Measured value (mg/kg)	
K	0.04	10,230 ± 640	9868.63 ± 480.71	96.5
Na	0.01	2033 ± 64	2086.09 ± 69.23	102.6
Ca	0.22	131 ± 10	140.35 ± 6.63	107.1
Mg	0.47	620.42 ± 42	627.05 ± 42.06	101.1
Zn	0.01	181.10 ± 1.00	176.11 ± 4.54	97.2
Fe	0.09	197.94 ± 0.65	196.51 ± 2.09	99.8
Mn	0.01	10.46 ± 0.47	10.49 ± 0.71	100.3
Cu	0.01	275.20 ± 4.60	287.19 ± 7.02	104.4

### Statistical Analysis

The statistical analyses were performed using SAS Enterprise Guide 6.1 software (SAS Institute Inc.). To test the normality and the homogeneity of variance of data, Kolmogorov–Smirnov’s test and Levene’s *F* test, respectively, were used. One-way analysis of variance (ANOVA), followed by Tukey’s (HSD) test, was used to compare means of mineral concentration in the muscles (LL vs. ST) or in the meat of different cattle breeds (PR, WB, PBW, SIM and PHF). Two-way ANOVA (with GLM procedure) was also employed to determine if interactions exist. Differences between means at confidence levels of 95 and 99 % (*P* < 0.05 and *P* < 0.01, respectively) were considered statistically significant. In Table 2, the mean value, standard error and range were presented.

**Table 2:** Statistical *P* values from the two-way ANOVA for breed and muscle and their interactions

Effect tested	Breed	Muscle	Interaction
K	**	NS	**
Na	**	NS	NS
Mg	**	NS	NS
Ca	**	NS	NS
Zn	**	*	NS
Fe	NS	**	NS
Mn	**	NS	**
Cu	**	**	NS

*NS* not significant

*P < 0.05; **P < 0.01

## Results and Discussion

The study showed that breed had a significant effect on the content of seven out of eight evaluated minerals and the type of muscle on the content of three elements. The significant interaction of both factors (breed x muscle) was found only for K and Mn (Table 2).

According to Cabrera et al. [1] and Ramos et al. [5], the most important factors influencing the proportions of minerals in cattle meat include breed, age, diet and production system, as well as the meat processing.

Our study showed that the meat of the PHF bulls contained significantly less K, Mg and Ca than beef of other breeds analysed (Table 3). The reverse tendency was observed in the case of the microminerals. The meat of the PHF bulls showed the highest content of most Mn (*P* < 0.01) and Fe (*P* > 0.05), as well as significantly more Zn than in the PR and SIM breeds (*P* < 0.01). The meat of the WB bulls contained significantly (*P* < 0.01) more Ca and less Na than the meat of the PR, SIM and PHF breeds. Moreover, the meat of WB bulls showed a significantly (*P* < 0.01) higher level of Mn and Cu in comparison with other native breeds (PR and PBW) and the SIM breed.

**Table 3:** Content of macro- and microminerals (in milligram per kilogram of fresh tissue) in the meat of young bulls depending on the breed and muscle (mean ± standard error and range in parentheses)

	Breed					Muscle	
	PR (*n* = 16)	WB (*n* = 16)	PBW (*n* = 16)	SIM (*n* = 16)	PHF (*n* = 16)	LL (*n* = 40)	ST (*n* = 40)
K	3838.03^C^ ± 68.75	3764.26^BC^ ± 95.72	3598.13^BC^ ± 77.75	3457.50^B^ ± 58.85	3204.80^A^ ± 63.89	3566.06 ± 57.14	3579.03 ± 66.84
	(3410.80–4346.09)	(3182.41–4355.53)	(3054.49–4169.20)	(2984.27–3903.80)	(2519.70–3678.26)	(2738.94–4355.53)	(2519.70–4346.09)
Na	483.55^B^ ± 37.09	352.64^A^ ± 11.22	414.94^AB^ ± 23.60	509.92^B^ ± 15.11	471.18^B^ ± 25.70	462.30 ± 18.30	430.59 ± 16.36
	(278.61–795.05)	(299.52–450.43)	(303.73–632.42)	(444.96–644.51)	(305.20–683.04)	(299.52–795.05)	(278.61–644.51)
Mg	319.49^B^ ± 4.83	299.10^B^ ± 8.59	320.90^B^ ± 7.35	312.54^B^ ± 7.60	236.88^A^ ± 7.37	297.49 ± 5.96	298.07 ± 7.45
	(280.49–361.91)	(245.14–345.65)	(297.46–381.65)	(259.65–364.56)	(175.05–276.80)	(203.54–381.65)	(175.05–376.93)
Ca	35.42^B^ ± 1.69	43.94^C^ ± 2.36	41.65^BC^ ± 2.84	33.58^B^ ± 0.97	20.41^A^ ± 2.10	37.12 ± 1.99	32.88 ± 1.63
	(25.69–49.10)	(26.27–56.10)	(30.29–66.64)	(26.55–39.30)	(11.90–40.35)	(11.90–66.64)	(12.43–55.76)
Zn	30.58^AB^ ± 20.56	36.81^BC^ ± 27.88	38.01^C^ ± 25.16	25.69^A^ ± 13.45	41.17^C^ ± 31.34	36.69^y^ ± 1.25	32.81^x^ ± 1.35
	(20.56–43.26)	(27.88–56.16)	(25.16–47.57)	(13.45–34.54)	(31.34–56.07)	(20.72–56.16)	(13.45–47.61)
Fe	21.36 ± 1.57	19.56 ± 0.85	18.81 ± 1.24	17.66 ± 2.02	21.77 ± 1.26	21.78^Y^ ± 0.88	17.88^X^ ± 0.87
	(12.21–30.58)	(14.12–27.44)	(11.62–27.83)	(6.58–32.19)	(11.05–27.93)	(12.00–32.19)	(6.58–27.93)
Mn	0.13^A^ ± 0.01	0.36^B^ ± 0.11	0.12^A^ ± 0.01	0.15^A^ ± 0.01	0.59^C^ ± 0.06	0.29 ± 0.05	0.23 ± 0.04
	(0.10–0.19)	(0.11–1.45)	(0.08–0.17)	(0.09–0.19)	(0.21–0.86)	(0.09–1.45)	(0.07–0.86)
Cu	0.55^A^ ± 0.03	0.71^B^ ± 0.03	0.55^A^ ± 0.02	0.52^A^ ± 0.03	0.59^AB^ ± 0.06	0.63^Y^ ± 0.02	0.54^X^ ± 0.02
	(0.34–0.80)	(0.56–0.93)	(0.46–0.70)	(0.30–0.70)	(0.30–0.96)	(0.41–0.96)	(0.30–0.78)

Means for breeds denoted by different letters in rows differ statistically significantly: A, B, C *P* < 0.01. Means for muscles denoted by different letters in rows differ statistically significantly: X, Y *P* < 0.01; x, y *P* < 0.05

The results of this study are similar to those obtained earlier by Florek et al. [9] for most of the minerals (K, Mg, Ca, Zn and Fe) determined in the muscles of young PHF bulls raised in eastern Poland.

The latest literature data also confirm that breed is a significant factor determining the content of minerals in the muscles of cattle raised under the same conditions [1, 5, 10]. Holló et al. [11] found significantly lower content of Ca and Na in the LL muscle of young bulls of the breed Hungarian Grey than in Holstein-Friesians. In the case of Mg, K, P, Cu, Zn and Fe, the differences were not confirmed statistically. Ramos et al. [5] analysed the content of microminerals (Se, Cu, Zn, Fe and Mn) in the muscles of Hereford and Braford steers and found significantly higher content of Cu and lower content of Fe in the former. On the other hand, Cabrera et al. [1] noted significant differences in steers of these breeds only in the case of Mn.

Leheska et al. [12], in strip steaks from pasture-raised cattle, found similar content of K, Fe Zn and Cu, higher Na and Ca and lower Mg and Mn than were found in the present study. Łozicki et al. [13] observed a higher level of Na, Zn and Fe in the meat of young bulls raised on an organic farm (with a diet mainly based on pasture forage) than in animals fed on maize silage and concentrate feed. On the other hand, Holló et al. [11], in the LL muscle of young bulls fed extensively (green forage/haylage with concentrate feed and flax), demonstrated lower content of Na and Cu and a higher level of Fe than in intensively fed young bulls (maize silage and concentrate feed).

Pilarczyk [10] found that breed had a significant effect on the content of K, Mg, Fe, Zn, Cu and Mn in the meat of intensively fed young bulls of the breeds Charolais, Hereford and Simmental raised in northwestern Poland. According to the author, the differences observed between the breeds were determined by metabolism. These differences may have been determined not only genetically but also by excretion of endogenous Cu and by the amount of fodder consumed by the animals.

The present study showed that while the type of muscle did not affect the content of macrominerals, in the case of microminerals, the effect was significant. Significantly higher content of Zn, Fe and Cu were noted in the LL muscle than in the ST (Table 3). Similarly, Florek et al. [14] demonstrated that the LL and ST muscles of two categories of PHF cattle differed significantly only for content of Zn, Mn and Cu. Ramos et al. [5] also found a significant influence of the muscle of steers on content of Se, Zn, Mn and Fe. The differences observed between muscles in the content of minerals may be linked to differences in their metabolic activity, fat content and blood circulation between organs [15, 16].

## Conclusions

The breed of cattle was found to affect the content of K, Na, Mg, Ca, Zn, Mn and Cu in the meat. The meat of the young PHF bulls contained smaller amounts of macrominerals (K, Mg and Ca) and larger amounts of microminerals (Zn, Fe and Mn) in comparison with the four other breeds.

Irrespective of the differences found, the meat of the cattle breeds evaluated is a valuable raw material containing nutritionally beneficial amounts of minerals, particularly Zn, Fe, Mg, Cu and K, as well as of Na (due to its low level). However, further research should be conducted both to determine the content of nutrients in other muscles/meat cuts and to assess the effect of various culinary procedures on their preservation and bioavailability.
